# A dynamically stable self-healable wire based on mechanical–electrical coupling

**DOI:** 10.1093/nsr/nwae006

**Published:** 2024-01-04

**Authors:** Shuo Wang, Zhaofeng Ouyang, Shitao Geng, Yan Wang, Xiaoju Zhao, Bin Yuan, Xiao Zhang, Qiuchen Xu, Chengqiang Tang, Shanshan Tang, Han Miao, Huisheng Peng, Hao Sun

**Affiliations:** Frontiers Science Center for Transformative Molecules, School of Chemistry and Chemical Engineering, Zhangjiang Institute for Advanced Study, and Key Laboratory of Green and High-End Utilization of Salt Lake Resources (Chinese Academy of Sciences), Shanghai Jiao Tong University, Shanghai 200240, China; Frontiers Science Center for Transformative Molecules, School of Chemistry and Chemical Engineering, Zhangjiang Institute for Advanced Study, and Key Laboratory of Green and High-End Utilization of Salt Lake Resources (Chinese Academy of Sciences), Shanghai Jiao Tong University, Shanghai 200240, China; Frontiers Science Center for Transformative Molecules, School of Chemistry and Chemical Engineering, Zhangjiang Institute for Advanced Study, and Key Laboratory of Green and High-End Utilization of Salt Lake Resources (Chinese Academy of Sciences), Shanghai Jiao Tong University, Shanghai 200240, China; Frontiers Science Center for Transformative Molecules, School of Chemistry and Chemical Engineering, Zhangjiang Institute for Advanced Study, and Key Laboratory of Green and High-End Utilization of Salt Lake Resources (Chinese Academy of Sciences), Shanghai Jiao Tong University, Shanghai 200240, China; Frontiers Science Center for Transformative Molecules, School of Chemistry and Chemical Engineering, Zhangjiang Institute for Advanced Study, and Key Laboratory of Green and High-End Utilization of Salt Lake Resources (Chinese Academy of Sciences), Shanghai Jiao Tong University, Shanghai 200240, China; Frontiers Science Center for Transformative Molecules, School of Chemistry and Chemical Engineering, Zhangjiang Institute for Advanced Study, and Key Laboratory of Green and High-End Utilization of Salt Lake Resources (Chinese Academy of Sciences), Shanghai Jiao Tong University, Shanghai 200240, China; Frontiers Science Center for Transformative Molecules, School of Chemistry and Chemical Engineering, Zhangjiang Institute for Advanced Study, and Key Laboratory of Green and High-End Utilization of Salt Lake Resources (Chinese Academy of Sciences), Shanghai Jiao Tong University, Shanghai 200240, China; Frontiers Science Center for Transformative Molecules, School of Chemistry and Chemical Engineering, Zhangjiang Institute for Advanced Study, and Key Laboratory of Green and High-End Utilization of Salt Lake Resources (Chinese Academy of Sciences), Shanghai Jiao Tong University, Shanghai 200240, China; State Key Laboratory of Molecular Engineering of Polymers, Department of Macromolecular Science, Institute of Fiber Materials and Devices, and Laboratory of Advanced Materials, Fudan University, Shanghai 200438, China; Frontiers Science Center for Transformative Molecules, School of Chemistry and Chemical Engineering, Zhangjiang Institute for Advanced Study, and Key Laboratory of Green and High-End Utilization of Salt Lake Resources (Chinese Academy of Sciences), Shanghai Jiao Tong University, Shanghai 200240, China; School of Materials Science and Engineering, East China University of Science and Technology, Shanghai 200433, China; State Key Laboratory of Molecular Engineering of Polymers, Department of Macromolecular Science, Institute of Fiber Materials and Devices, and Laboratory of Advanced Materials, Fudan University, Shanghai 200438, China; Frontiers Science Center for Transformative Molecules, School of Chemistry and Chemical Engineering, Zhangjiang Institute for Advanced Study, and Key Laboratory of Green and High-End Utilization of Salt Lake Resources (Chinese Academy of Sciences), Shanghai Jiao Tong University, Shanghai 200240, China

**Keywords:** self-healable wire, interfacial chemistry, polymer materials, mechanical–electrical coupling, fiber device

## Abstract

The rise in wearable electronics has witnessed the advancement of self-healable wires, which are capable of recovering mechanical and electrical properties upon structural damage. However, their highly fluctuating electrical resistances in the range of hundreds to thousands of ohms under dynamic conditions such as bending, pressing, stretching and tremoring may seriously degrade the precision and continuity of the resulting electronic devices, thus severely hindering their wearable applications. Here, we report a new family of self-healable wires with high strengths and stable electrical conductivities under dynamic conditions, inspired by mechanical–electrical coupling of the myelinated axon in nature. Our self-healable wire based on mechanical–electrical coupling between the structural and conductive components has significantly improved the electrical stability under dynamic scenarios, enabling precise monitoring of human health status and daily activities, even in the case of limb tremors from simulated Parkinson's disease. Our mechanical–electrical coupling strategy opens a new avenue for the development of dynamically stable electrodes and devices toward real-world wearable applications.

## Teaser

Self-healable wires with high strength and stable electrical conductivity under dynamic conditions have been developed based on a novel mechanical-electrical coupling effect, which show great potential for practical applications in highly dynamic scenarios.

## INTRODUCTION

Highly flexible, strong and conductive wearable wires are crucial for reliable interconnection of wearable devices [1–4]. However, the frequent and diverse deformations in practical use often lead to structural damage of these wires, resulting in decreased lifespan or even the failure of the entire module [5–8]. Self-healable wires, which can recover their mechanical and electrical properties upon structural damage, provide a promising solution to solving these issues [9–20]. However, the practical applications of self-healable wires have been severely hindered by two key challenges. On the one hand, their tensile strengths (0.05–11 MPa) are generally much lower than those of common textile fibers [21,22], such as cotton (28–49 MPa), polyamide (PA) (45–73 MPa) and polyethylene terephthalate (PET) (60–74 MPa), resulting in severe mechanical mismatch and structural instability for the weaving and wearing processes. More importantly, the fluctuating electrical resistances of these wires under dynamic conditions such as bending, pressing, stretching and tremoring have severely reduced the precision and reliability of the interconnected wearable devices in practical applications [23–28]. The key challenge lies in the fragile interface between the structural and conductive components, which leads to inferior mechanical performance and dynamic stability of these self-healable wires.

**Figure 1.**
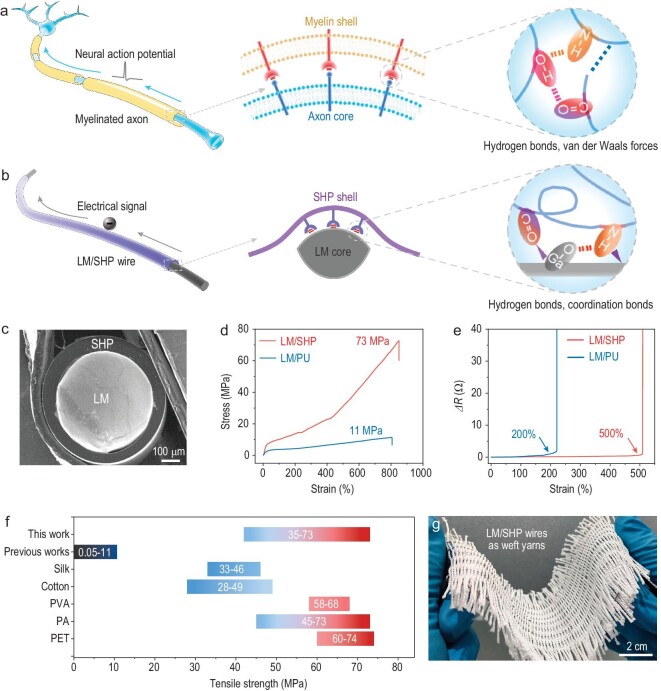
Dynamically stable self-healable LM/SHP wires inspired by the myelinated axon. (a) Schematic illustration of the core–shell configuration of the myelinated axon. The magnified image illustrated the interactions contributed by hydrogen bonds and Van der Waals forces between the myelin shell and the axon core. (b) Schematic illustration of our LM/SHP wires. The magnified image illustrated the hydrogen-bond and coordination-bond interactions between the SHP shell and the LM core. (c) Cross-sectional SEM image of the LM/SHP wire. (d) Tensile stress–strain curves of the LM/SHP and LM/PU wires. (e) Variation in the electrical resistance of LM/SHP and LM/PU wires during stretching. (f) Comparison of the tensile strengths of our LM/SHP wire, the previously reported self-healable wires and the common textile fibers including silk, cotton, polyvinyl alcohol, polyamide (PA) and polyethylene terephthalate (PET) [9–22]. (g) LM/SHP wires (weft yarns) were woven with PET fibers (warp yarns) to form a flexible and breathable fabric.

In our attempts to address these issues, we recognize that myelinated axon—the essential function unit of the nervous system—might be an inspiring model [29,30]. The myelin shell on the outer surface of the myelinated axon serves as a structural component for protection of the inner axon, while the inner axon serves as the pathway for saltatory conduction of neural action potential (Fig. 1a). The interaction between the myelin and axon, based on the hydrogen bonds and Van der Waals forces, guarantees reliable transmission of neural action potential under diverse deformations, which underlies various biological behaviors in nature [31,32]. This has inspired us to design the interaction between the structural and conductive components of self-healable wires to solve the above-mentioned challenges.

Here, we develop a new family of self-healable wires with excellent dynamic stability inspired by the mechanical–electrical coupling of myelinated axon. Our self-healable wire is composed of a self-healable polymer (SHP) as the robust and flexible shell and a GaInSn liquid metal (LM) as the conductive core. Together, they yield an exceptional tensile strength of 73 MPa at a strain of 850% and an electrical conductivity of 9.8 × 10^4^ S m^–1^ with impressive self-healable capabilities. The mechanical–electrical coupling based on hydrogen and coordination bonds between the SHP and the LM has significantly enhanced the dynamic stability of the self-healable wires, achieving ultra-stable electrical resistances under a high strain of 500%. As a proof of concept, we used these self-healable wires for interconnection of numerous electronic devices into integrated healthcare modules for precise monitoring of human health status and daily activities, even in the case of limb tremors from simulated Parkinson's disease. These dynamically stable self-healable wires can benefit a wide range of applications such as wearable healthcare, intelligent robotics and implantable electronics.

## RESULTS AND DISCUSSION

The SHPs were synthesized via a two-step condensation polymerization method (Fig. S1). The hard segments of the polymer chains were optimized through rational regulation of the hydrogen-bond domains of acyl-semicarbazide (ASC) moieties and the poly(1,4-butylene adipate) served as a soft segment to bridge the hard segments. This not only interlocked the polymer chains, but also allowed effective energy dissipation, which together contributed to high tensile strengths and toughness [33,34]. SHP with a crosslink density (*χ*) of 0.18 (named SHP-0.18) showed a tensile strength of 80 MPa at a strain of 1017%, corresponding to a toughness of 337 MJ cm^–3^ (Fig. S2a and Table S1). A mechanical healing efficiency of 76% could be achieved via the dynamic reversibility of the abundant hydrogen bonds and ASC moieties (Figs S2b and c, and S3). Thermogravimetric analysis showed the high thermal stability of SHP-0.18 with 5% weight loss at <310°C (Fig. S2d). The dynamic property of the ASC moiety was verified by using ^1^H nuclear magnetic resonance (^1^H NMR) and gel permeation chromatography (GPC). The dynamic exchange between 2-benzoyl-*N*-hexylhydrazine-1-carboxamide and acethydrazide was observed in ^1^H NMR with the formation of two new types of products including benzoylhydrazine and 2-acetyl-*N*-hexylhydrazine-1-carboxamide (Figs S4 and S5a and b). The increased retention time in GPC indicated the decreased molecular chain length of SHP due to the dynamic dissociation-exchange reaction between SHP and 2-acetyl-*N*-hexylhydrazine-1-carboxamide (Fig. S5c and d), proving the dynamic property of ASC moieties in polymer chains.

As mentioned above, the interaction between the core–shell components of the myelinated axon ensures reliable transmission of the neural action potential under dynamic conditions (Fig. 1a), which provides an inspiring model for the development of self-healable wires with high dynamic stability. Specifically, the optimized SHP was employed as the robust shell coupled with GaInSn LM as the conductive core (Fig. 1b). The intimate interface between the structural (SHP) and conductive (LM) components was verified by using scanning electronic microscopy (SEM) (Fig. 1c and Fig. S6). The LM/SHP wire exhibited a high tensile strength of 73 MPa at a strain of 850% based on the abundant hydrogen bonds of the ASC moieties (Fig. 1d). As a comparison, polyurethane (PU) synthesized with fewer hydrogen bonds by replacing isophthalic dihydrazide with 1,3-dihydroxybenzene exhibited a much lower tensile strength of 11 MPa (Fig. 1d and Fig. S1) and polydimethylsiloxane (PDMS) and polyethylene (PE) also showed lower tensile strengths of 5 and 12 MPa, respectively (Fig. S7a). The tensile strengths of LM/SHP wires could be further regulated by varying the crosslink density of SHP, realizing tensile strengths of 35–73 MPa, which showed a good mechanical match with common textile fibers (28–74 MPa) (Fig. 1f, Fig. S8 and Tables S2 and S3) [21,22]. In addition, the Young's moduli of 75–86 MPa were lower than those of common textile fibers (81–851 MPa), indicating good flexibility and fitness for the human body, and the higher strains (at 850–1014%) compared with the 15–58% of common textile fibers could be of benefit in high-strain applications (Table S3).

Cyclic tensile tests were further carried out to investigate the elastic recovery properties of the LM/SHP wire, which showed obvious hysteresis loops at different strains from 50% to 400%, indicating the substantial energy dissipation attributed to the force-induced rupture of the abundant hydrogen bonds (Fig. S9a). An extended cyclic tensile test was also performed with 50% and 100% strains for 150 cycles, which exhibited a gradual increase in residual strain (Fig. S9b and c). Impressively, the cyclic tensile curves and hysteresis loops could almost recover to the original state after thermal treatment at 80°C for 1 min (Fig. S9d), which involved polymer chain re-entanglement and hydrogen-bond recombination [35,36].

Practical wearable applications require self-healable wires with stable electrical resistances under diverse deformations, which remains a key challenge for the whole field [24,28]. For instance, the composite wires (0.7 and 20 cm in diameter and length, respectively) based on PU, PDMS and PE exhibit relatively large resistance increases of 1.0–65.2 Ω at a strain of 200% (Fig. 1e and Fig. S7b). In comparison, our LM/SHP wire showed slight resistance increases of 0.1 and 0.7 Ω at high strains of 200% and 500%, respectively (Fig. 1e). Notably, the high strain of 500% might not represent a practical condition for human wearable applications, but might be useful for elastic actuators and artificial robots that require high strains. To further evaluate the stability and durability of LM/SHP wire, a cyclic tensile test with different strains from 50% to 400% was conducted (Fig. S10a and b). It showed an excellent electrical stability with a small *R/R*_0_ value of <1.06 even at a high strain of 400%. The extended cyclic tensile test further illustrated the high stability of our LM/SHP wire, which exhibited small *R/R*_0_ values of 1.06 at 50% strain for 450 cycles and 1.05 at 400% strain for 50 cycles (Fig. S10c and d) [20]. A high electrical conductivity of 9.8 × 10^4^ S m^–1^ could be achieved by using an LM/SHP wire with a diameter and length of 0.7 and 5 cm, respectively (Equation S1). Impressively, a 20-cm-long LM/SHP wire could be stretched to 1 m when heated to 100^o^C and maintained the stretching state when cooling down to room temperature (Figs S11 and S12), presenting a facile approach for regulating wire lengths. As a demonstration, the LM/SHP wires were used as weft yarns woven with PET warp yarns to form a fabric with high flexibility and breathability (Fig. 1g).

**Figure 2.**
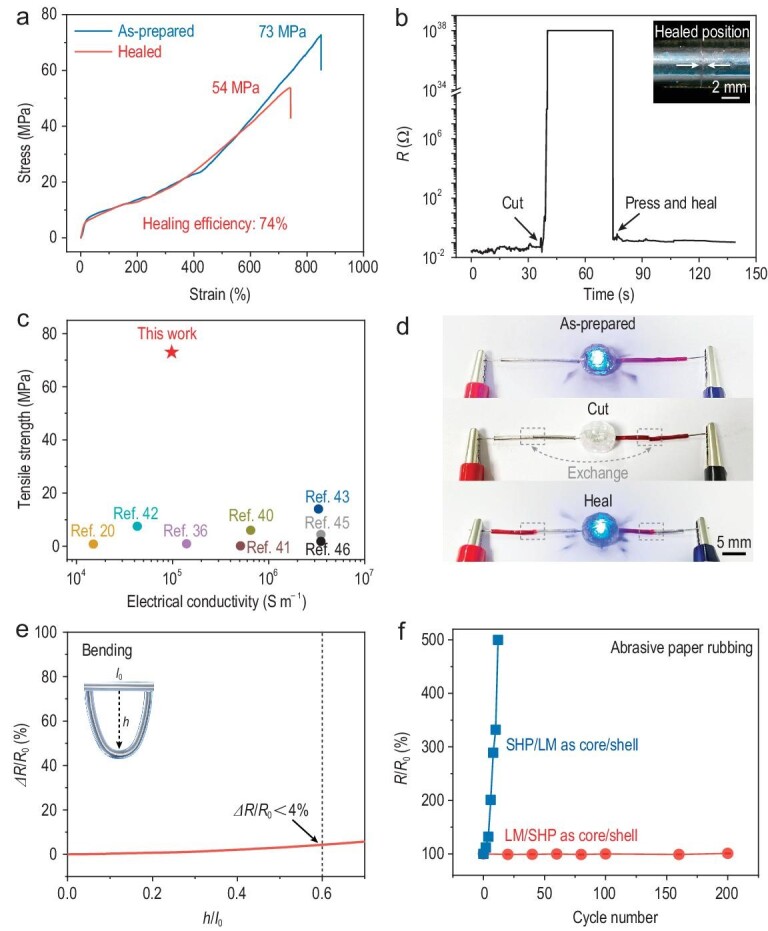
Mechanical, electrical and self-healing properties of the LM/SHP wires. (a) Tensile stress–strain curves of the LM/SHP wires with and without the breaking and healing processes. (b) Variation in the electrical resistances of an LM/SHP wire during the breaking and healing processes. The inset shows the morphology of the healed position. (c) Comparison of the electrical conductivities and tensile strengths of the LM/SHP wires with previously reported core–shell LM-based fibers [20,36,40–43,45,46]. (d) Two colored LM/SHP wires connected in a circuit containing an LED lamp and two coin cells before and after breaking and healing. (e) Variation in the electrical resistances of LM/SHP wires during the bending test. (f) Comparison of the electrical resistance changes of LM/SHP and SHP/LM wires during repeated rubbing on abrasive paper.

The LM/SHP wires exhibited impressive self-healing performance in both mechanical and electrical properties. In previous works, the dynamic dissociation-exchange reaction of ASC moieties has been confirmed [33,37] and we showed that ASC moieties could be reversibly cleaved into isocyanate and hydrazide groups by using ^1^H NMR and GPC (Figs S3 and S5). The self-healing properties of LM/SHP wires were carefully investigated under different healing conditions (Figs S3a and Table S4). For instance, the healed tensile strength could reach 54 MPa (74% of the original value) after complete breaking and healing at 110°C for 12 h (Fig. 2a). Decreasing the thermal treatment time led to lower healing efficiencies due to the time-dependent recovery of dynamic covalent networks [38]. Increasing the healing temperature (e.g. 120°C for 12 h) would slightly decrease the healing efficiency. A content increase in the LM would result in a tensile strength of 67 MPa and healing efficiency of 77% (Fig. S13b).

In addition, the electrical resistance of a 5-cm-long LM/SHP wire (0.7 mm in diameter) increased from 0.013 to 10^38^ Ω after complete cutting-off (Fig. 2b). After healing, the electrical resistance almost recovered to the original level, owing to the fusion of the LM that rebuilt the conductive pathway. There could be some loss of the LM during the breaking and healing processes due to its fluidic nature, which might result in a slight resistance increase after healing. Slight scratches were observed at the breaking/healing position without observable gaps or voids (Fig. S14). Notably, the healed LM/SHP wire exhibited small resistance fluctuations of <6.5% after stretching for 500 cycles at a strain of 50% (Fig. S15). LM has been widely used for preparation of core–shell wires in previous studies [18,20,36,39–46]. To the best of our knowledge, our LM/SHP wire exhibited a higher tensile strength (73 MPa) and competitive electrical conductivity (9.8 × 10^4^ S m^–1^) over the other core–shell LM-based fibers (Fig. 2c and Table S7) [20,36,40–43,45,46]. The healed tensile strength (54 MPa) and electrical conductivity (1.2 × 10^4^ S m^–1^) are among the best metrics of state-of-the-art self-healable wires (Fig. S16 and Table S6) [9,12–17,19]. As a demonstration, a light-emitting diode (LED) lamp and two coin cells were connected using two colored LM/SHP wires, which were completely cut off and healed (Fig. 2d). The brightness of the LED lamp remained almost the same after healing, suggesting effective recovery of the electrical property.

The mechanical and electrical properties of the LM/SHP wires were further investigated under various dynamic conditions such as bending, pressing, knotting and twisting. During the bending test with a harsh *h/l_0_* ratio of 0.6, the electrical resistance was increased by <4% (Fig. 2e). Under pressure of 11 MPa, the LM/SHP wire showed a slight electrical resistance fluctuation of <2% (Fig. S17a). In addition, the electrical resistance could be stably maintained under harsher deformations such as knotting and twisting (Fig. S17b). The washing test verified a slight resistance variation within 5% after 2 h of washing (Fig. S18a and b). The tensile strengths remained almost unchanged after drying as well (Fig. S18c). We also investigated the advantages of the core–shell configuration of our LM/SHP wires, i.e. SHP as the core and LM as the shell. Our LM/SHP (core/shell) wire showed negligible electrical resistance variations when rubbed repeatedly on abrasive paper for 200 cycles (Fig. 2f), while the SHP/LM (core/shell) wire displayed a 500% increase in electrical resistance after only 12 cycles. These results verified the effective protection of the SHP shell for the inner LM core, as well as the mechanical–electrical coupling between the structural and conductive components.

**Figure 3.**
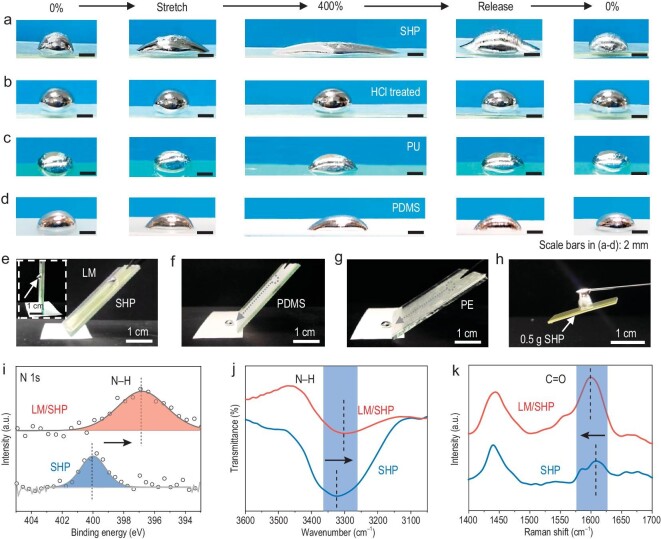
Mechanism study of mechanical–electrical coupling. (a and b) Variation in the contact angles of an LM droplet on the SHP substrate under different strains before and after dilute hydrochloric acid treatment, respectively. (c and d) Variation in the contact angles of an LM droplet on the PU and PDMS substrate under different strains. The PDMS substrate was directly used without oxygen plasma treatment. (e–g) Photographs of LM droplets on SHP, PDMS and PE substrates in the obliquity experiment. (h) Photograph of an LM droplet lifting a SHP board based on the strong interaction between them. The weights of the LM droplet and the SHP board were both 0.5 g. (i) High-resolution XPS spectra for N 1s of bare SHP and the LM/SHP composite. The N 1s peak significantly shifted to a lower binding energy owing to the interaction between the SHP and the LM. (j) FTIR spectra of bare SHP and the LM/SHP composite in the N–H stretching region. (k) Raman spectra of bare SHP and LM/SHP composite in the C=O stretching region. The mass ratio of the LM/SHP in (i–k) was 1 : 1.

The mechanical–electrical coupling between the SHP and the LM has significant implications for the dynamic stability of the self-healable wires. In an experiment for analysis of the interface interaction, we tracked the contact angles of an LM droplet on an SHP substrate during stretching. The LM was simultaneously stretched along the stretching direction of the SHP with close contact even at a strain of 400% and it could recover to its original shape after releasing (Fig. 3a), suggesting the strong interaction between the LM and the SHP. As a comparison, when the oxidation layer was removed by using dilute hydrochloric acid, the LM droplet remained in the spherical shape throughout the stretching and releasing processes (Fig. 3b), indicating the significant influence of the oxidation layer on the LM [42,47]. In comparison, PU and PDMS exhibited negligible changes in contact angles under the same condition, revealing their weak interactions with the LM (Fig. 3c and d). We further performed the obliquity experiment and the LM droplet could not roll off the SHP substrate even at an inclination of 90° due to the strong interfacial interaction (Fig. 3e). In contrast, the LM droplet could rapidly roll off the PDMS and PE substrates at an inclination angle of <60^o^ (Fig. 3f and g). Impressively, a 0.5-g LM droplet could lift an SHP board of equivalent weight (Fig. 3h). These results confirmed the presence of strong interfacial interactions between the SHP and the LM, contributing to the mechanical–electrical coupling for achievement of high dynamic stability.

We further performed X-ray photoelectron spectroscopy (XPS), Fourier transform infrared spectroscopy (FTIR) and Raman spectroscopy to probe the interfacial interactions between the SHP and the LM. The coexistence of Ga_2_O_3_ and hydroxyl groups on the surface of the LM was verified by using the high-resolution Ga 2p and O 1s spectra of XPS (Fig. S19), derived from spontaneous oxidization of the liquid Ga metal in an air atmosphere [48–50]. In addition, the N–H peak of the N 1s spectra at 399.9 eV shifted to 396.8 eV after the incorporation of the LM, suggesting the strong interaction between the N–H function groups and the Ga_2_O_3_ layer (Fig. 3i) [51,52]. The N–H stretching vibration at 3323 cm^–1^ in the FTIR spectra shifted to a lower wave number of 3301 cm^–1^ after LM incorporation (Fig. 3j), which revealed the formation of a hydrogen bond between the N–H and Ga_2_O_3_ layer [53]. The red shift of the N–H group after Ga^3+^ incorporation was attributed to the formation of the coordination bond between the N–H and the Ga^3+^, which liberated some N–H groups that were previously bound with C=O by hydrogen bonding (Fig. S20) [38]. The introduction of the LM also shifted the C=O stretching vibration to a lower frequency (from 1608 to 1599 cm^–1^) in Raman spectra, indicating the presence of coordination bonds between the C=O groups in the SHP and Ga cations in the LM (Fig. 3k) [54,55]. Therefore, the mechanical–electrical coupling can be attributed to the hydrogen bonds between the N–H (in the SHP) and the Ga_2_O_3_ (LM surface) and the coordination bonds between the C=O, N–H groups (in the SHP) and Ga cations (in the LM), which sufficiently enhanced the interactions between the structural (SHP) and conductive (LM) components (Fig. S21), thus achieving high electrical stability even under high strains.

**Figure 4.**
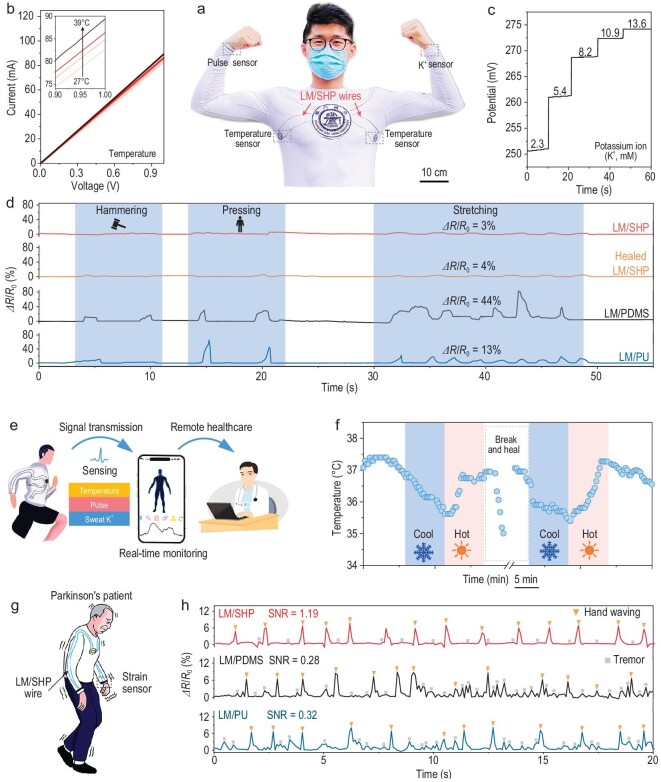
Wearable applications of the dynamic-stable and self-healable LM/SHP wire. (a) Photograph of an integrated wearable human-healthcare platform consisting of three wearable sensors (temperature, pulse and K^+^), a microcontroller unit, a Bluetooth module and a lithium-ion battery. The LM/SHP wires were used for connection of all the components. (b) Current–voltage curves of the wearable temperature sensor at different temperatures from 27^o^C to 39^o^C, which exhibited a good linear relationship between the current and the voltage. (c) The open-circuit potential responses of the K^+^ sensor in potassium chloride solutions with varying concentrations. (d) The resistance changes of LM/SHP, healed LM/SHP, LM/PDMS and LM/PU wires under dynamic scenarios such as hammering, pressing and stretching. (e) Schematic illustration of the wearable healthcare platform with real-time monitoring and remote healthcare capabilities. (f) Real-time temperature monitoring of the human body using the wearable healthcare platform based on LM/SHP wires. After the breaking and healing of the LM/SHP wire, the wearable temperature sensor could work normally. (g) Schematic illustration of hand-waving signal detection using a strain sensor attached to the wrist of a patient with Parkinson's disease. The LM/SHP wires were used for connecting the strain sensor and the microcontroller unit. (h) Detection profiles of the strain sensors based on LM/SHP, LM/PDMS and LM/PU wires. The signal-to-noise ratios (SNRs) of LM/SHP, LM/PDMS and LM/PU wires were 1.19, 0.28 and 0.32, respectively.

The appealing mechanical, electrical and dynamic properties of our LM/SHP wires make them promising candidates in wearable applications. As a proof of concept, we fabricated an integrated healthcare platform consisting of multiple sensors (e.g. temperature, pulse and K^+^), a microcontroller unit (MCU), a Bluetooth module and a lithium-ion battery. These components were connected using our LM/SHP wires (Fig. 4a and Figs S22 and S23). All the sensors exhibited reliable performance interconnected with the LM/SHP wires, even after breaking and healing of the LM/SHP wire (Fig. 4b and c, and Fig. S24). Notably, our LM/SHP wires exhibited a negligible electrical resistance fluctuation of 3–4% under various dynamic scenarios, including hammering, pressing and stretching, even after breaking and healing (Fig. 4d), which ensured stable and reliable operation of the interconnected wearable devices. In contrast, the wires based on PDMS and PU exhibited significant electrical resistance fluctuations of 13–44% under hammering, pressing and stretching, indicating inferior interfacial stability in the absence of mechanical–electrical coupling regulation. The high dynamic stability of our LM/SHP wire thus ensured reliable operation of the integrated human-healthcare platform, and the measured physiological data could be wirelessly transmitted to physicians and displayed on a mobile phone (Fig. 4e). For instance, the temperature sensor could track real-time temperature changes of the human body under different conditions (Fig. S25). Importantly, after the breaking and healing of the LM/SHP wire, the temperature sensor could work normally, which would improve the lifespan and reliability of the entire wearable healthcare platform (Fig. 4f).

In addition, we verified the reliable monitoring of hand-waving signals (∼0.7 Hz) under continuous limb tremor (∼2.7 Hz) using a strain sensor interconnected by our LM/SHP wires, which simulated the cases of limb tremors caused by Parkinson's disease (Fig. 4g and Fig. S26) [56,57]. Attributed to the high dynamic stability of our LM/SHP wire, a high signal-to-noise ratio (SNR) of 1.19 could be realized (Fig. 4h, Fig. S27 and Equation S4). In comparison, the strain sensor connected by LM/PDMS and LM/PU wires showed significant signal fluctuations with poor SNRs of only 0.28–0.32 under the same condition. It is worth noting that the complexity and variability of Parkinson's tremors in real-life scenarios could be significantly different from the conditions simulated in a controlled environment, which require further investigation and improvement in future studies to verify the practicability. These results confirmed the dynamic stability of our LM/SHP wire derived from mechanical–electrical coupling, making it a promising candidate for various wearable applications toward high reliability and stability.

## CONCLUSION

In summary, we report a new family of dynamically stable self-healable wires based on a mechanical–electrical coupling effect inspired by the myelinated axon in nature. Mechanical–electrical coupling between the SHP and the LM provides strong interactions based on the hydrogen bonds and coordination bonds, which significantly enhance the interfacial stability even under highly dynamic conditions. The obtained self-healable wires exhibit high tensile strengths that solve the mechanical mismatch with common textile fibers and demonstrate ultra-stable electrical properties under diverse dynamic conditions. As a proof of concept, we fabricated a highly reliable wearable healthcare platform consisting of multiple electronic devices interconnected using our self-healable wires. In addition, our self-healable wires allow precise monitoring of hand-waving signals under a case of limb tremors derived from simulated Parkinson's disease with a high SNR of 1.19. Our results not only represent an important step for practical applications of self-healable wires, but, in a broader context, also provide a new paradigm for synergistic improvement of the mechanical and electrical properties of wearable electrodes and devices, particularly under highly dynamic scenarios in practical applications.

## Supplementary Material

nwae006_Supplemental_File
