# Supplementary material for: A dynamically stable self-healable wire based on mechanical–electrical coupling
Source: Natl Sci Rev. 2024 Jan 4;11(3):nwae006. doi: 10.1093/nsr/nwae006 (PMC10858651; doi:10.1093/nsr/nwae006)
Supplement: nwae006_Supplemental_File [file nwae006_supplemental_file.pdf]

SUPPORTING INFORMATION

A dynamically stable self-healable wire based on mechanical-electrical coupling

Shuo Wang, Zhaofeng Ouyang, Shitao Geng, Yan Wang, Xiaoju Zhao, Bin Yuan, Xiao Zhang, Qiuchen Xu, Chengqiang Tang, Shanshan Tang, Han Miao, Huisheng Peng, and Hao Sun*

This file includes:

Experimental Section (Pages 2-5)
Supporting Figures (Pages 6-33)
Supporting Tables (Pages 34-41)
References (Page 42-43)

EXPERIMENTAL SECTION

Materials. Isophthalic dihydrazide (IPDH, 95.0%, TCI), 1,3-dihydroxybenzene (DHB, 98%, Adamas), hexamethylene diisocyanate (HDI, 98%, Adamas), hexyl isocyanate (98%, TCI), benzoylhydrazine (98%, Adamas), acethydrazide (98%, Adamas), hexamethylene diisocyanate trimer (tri-HDI, Desmodur N3300, Bayer), *N*,*N*-dimethylformamide (DMF, water content below 50 ppm, Adamas), tetrahydrofuran (THF, water content below 50 ppm, Adamas), poly(3,4-ethylenedioxythiophene)/poly(styrenesulfonate) (PEDOT:PSS, CLEVIOS™ PVP AI 4083, Heraeus), valinomycin (98%, Macklin), sodium tetraphenylborate (99%, Macklin), polyvinyl chloride (PVC, 99%, Adamas), bis(2-ethylehexyl) sebacate cyclohexanone (99%, Macklin), potassium nitrate (KNO_3, 99%, Adamas), silver nitrate ($AgNO_3$, 98%, Macklin), potassium chloride (KCl, 99%, Adamas), Nafion (99%, Aladdin), glutaraldehyde (99%, Aladdin), polytetrafluoroethylene (PTFE) threads (Hengteng Plastic Co., Ltd.), and carbon fiber (CF, Zhongfu Shenying Carbon Fiber Co., Ltd.) were used as received. Poly(1,4-butylene adipate) (PBA, M_n = 1,000 g mol^{-1}, Macklin) was dried under vacuum at 110 ℃ for 2 h prior to use. The GaInSn liquid metal (LM) used in this study was composed of 68.5 wt% Ga, 21.5 wt% In and 10 wt% Sn (99.999%, ρ=6.44 g cm^{-3} at 20 ℃, Hunan Xineng Materials Co., Ltd.) with a low melting point (10 ℃) and high electrical conductivity (3.46×10^6 S m^{-1} at 20 ℃). The carbon nanotube fibers (CNTs) were prepared *via* floating catalyst vapor deposition with ferrocene and thiophene as catalysts [1].

Synthesis of 2-benzoyl-N-hexylhydrazine-1-carboxamide. Benzoylhydrazine (10 mmol, 1.36 g) and hexyl isocyanate (10 mmol, 1.28 g) were dissolved in 10 mL THF and stirred for 2 h at 40 ℃. 2-benzoyl-*N*-hexylhydrazine-1-carboxamide was precipitated from the solution after refrigerating for 6 h at 0 ℃, and then collected by centrifugation and finally dried at 60 ℃ for 12 h.

Nuclear magnetic resonance (NMR) study of the ASC moiety dynamic property. 2-benzoyl-*N*-hexylhydrazine-1-carboxamide (0.2 mmol, 0.053 g) and acethydrazide (0.2 mmol, 0.015 g) were dissolved in 5 mL DMSO-d_6, and divided in 5 NMR tubes. The obtained 5 samples were reacted at 120 ℃ for 0, 5, 15, 21 and 35 h respectively, and then were characterized by ^{1}H NMR.

Gel permeation chromatography (GPC) study of the ASC moiety dynamic property in polymer chain. HDI (2 mmol, 0.34 g) and PBA (1 mmol, 1 g, M_n = 1,000 g mol^{-1}) were reacted for 12 h at 80 ℃ in DMF (10 mL). IPDH (1 mmol, 0.19 g) was added into the above solution and stirred for 1 h at room temperature to obtain linear self-healable polymer (SHP). The solution was divided into 5 glass bottles after

adding 2 mmol 2-benzoyl-*N*-hexylhydrazine-1-carboxamide. They were reacted for 0, 1, 2, 5 and 10 h at 120 ℃ respectively, then were performed on GPC.

Synthesis of SHPs. SHPs were synthesized *via* condensation polymerization between isocyanate, diol, hydrazide monomers, and crosslinkers. The obtained SHPs were named SHP-χ, where χ represented crosslink density (Equation S2). The typical synthesis process of SHP-0.18 was described as follows. First, the PBA-HDI pre-polymer was synthesized by condensation reaction between HDI (13.34 mmol, 2.24 g) and PBA (6.67 mmol, 6.67 g, M_n = 1000 g mol^{-1}) for 12 h at 80 ℃ in 5 mL DMF. Then 30 mL DMF, IPDH (8.34 mmol, 1.62 g) and tri-HDI (1.11 mmol, 0.56 g) were added into the pre-polymer solution, and stirred at room temperature for 1 h. The obtained solution was poured into Teflon mold, and dried at 80 ℃ for 48 h, followed by drying at 110 ℃ under vacuum condition for 48 h. Other SHPs with varying χ and polyurethane (PU, the molar ratio of monomers was consistent with SHP-0.18) with fewer hydrogen bonds were synthesized by using the same processes. All the samples were stored in a desiccator before using. The monomer ratios of SHPs were shown in Table S8 in details.

Preparation and healing processes of LM/SHP wires. The SHP shells were prepared by brush-coating and template methods. The SHPs were dissolved in DMF with a concentration of 0.33 g mL^{-1}. The obtained solution was uniformly coated on PTFE threads, followed by complete removal of solvent for 48 h at 110 ℃ under a vacuum condition. The inner diameters of the LM/SHP wires were controlled by varying the diameter of the PTFE threads, *e.g.*, 0.6, 1, and 1.5 mm, corresponding to increased LM content (Figure S28). For example, the LM/SHP wires with inner diameters 1.5 mm represented higher LM content. The PTFE threads were then drawn, obtaining a hollow tube for injection of the LM by using a syringe. The wires could be easily encapsulated by heating the both sides at 150 ℃ for 5 min based on the self-healing capability of SHPs. The SHP samples were cut in the middle to form a ~90% crack, and then healed for 12 h at 110 ℃. The LM/SHP wires were cut completely using a blade, and the interfaces at the fractured position required close contact. They were then healed for 1, 6, and 12 h at 110 or 120 ℃ in an oven. The healing efficiency was determined by the ratio of fracture strength before and after breaking-healing. At least three samples were tested. The healing performance was presented in Table S3 and Figure S13.

Preparation of the pulse, temperature, K^+, and strain sensors. The pulse, temperature, and strain sensors were fabricated *via* simple wrapping and curing packaging methods. Specifically, a polyurethane fiber was employed as supporting material with its two ends fixed by two clamps. Subsequently, the CFs were wrapped

on the surface of the polyurethane fiber, and the ultraviolet curing adhesive was coated on the surface as a protective layer. The obtained polyurethane/CF composite fibers were utilized as pulse, temperature, and strain sensors for integrated healthcare platform to monitor human health status and hand-waving signals, respectively. The K^+ sensor was prepared *via* a dip-coating method. The CNTs were selected to absorb PEDOT:PSS, and then coated a layer of K^+ selective membrane solution *via* dip-coating method, and dried at 25 ℃. The K^+ selective membrane solution was composed of 2 wt% valinomycin, 0.5 wt% sodium tetraphenylborate, 32.7 wt% polyvinyl chloride, 64.7 wt% bis(2-ethylehexyl) sebacate, and 350 μL cyclohexanone. Ag/AgCl reference electrode was manufactured by cyclic voltammetry method. The CNTs were electrodeposited a layer of Ag *via* sweeping from –0.9 V to 0.9 V for seven cycles at 0.1 V s^{-1} in a 5 mM $AgNO_3$/1 M KNO_3 solution. Then the obtained fibers were electrodeposited a layer of AgCl *via* sweeping from –0.15 V to 1.05 V for two cycles at 0.05 V s^{-1} in a 0.1 M KCl/0.01 M HCl solution. In order to eliminate the potential drift, a polymer layer consisted of Nafion and glutaraldehyde was finally coated onto the above fibers, thus obtaining fiber-shaped Ag/AgCl reference electrodes.

Production of the integrated healthcare module. A tight clothing was used to guarantee the close contact with the human body. Besides, different weaving processes were applied to facilitate the intimate contact between the sensors and human body. For the temperature and pulse sensors, they were sewn on the outer surface of tight clothing with a slight pressure of ~0.2 MPa applied to ensure the intimate contact. The K^+ sensor was woven on the inner surface of the tight clothing to enable intimate contact with the skin for better K^+ signal detection. The above sensors were connected with a microcontroller unit (MCU), a lithium-ion battery, and a Bluetooth module using LM/SHP wires as the connecting components, and assembled into a human-healthcare platform for real-time monitoring of pulse, body temperature, and K^+ concentration in sweat. For instance, upon the body temperature changes, polyurethane/CF composite fiber as temperature sensor captured the temperature signals and converted them into electrical signals for input to MCU. After data analysis, the relevant instructions were transferred to mobile phone for real-time display.

Characterizations. Scanning electron microscope (SEM) was conducted on a FEI-Quanta 250 field at an acceleration voltage of 5 kV. Tensile experiments were conducted on an Instron 3365 equipped with a 1 kN load cell at 25 ℃ with a tensile rate of 50 mm min^{-1}. The dimensions of SHP boards and LM/SHP wires were dumbbell-shaped (*ca.* 0.5 mm (T) × 2 mm (W) × 35 mm (L) and a gauge length of 15 mm) and fiber-shaped tensile samples (*ca.* 2.5-3 mm (D) × 35 mm (L) and a gauge

length of 20 mm), respectively. At least three samples were tested. Fourier transform infrared spectroscopy (FTIR) analysis was performed on a Nicolet 560 FTIR spectrometer. X-ray photoelectron spectroscopic (XPS) analysis was performed on a PHI 5000 Versaprobe III. Raman spectra was measured by a Via-Reflex Raman spectrometer (LabRAM Solei) with a laser at 532 nm wavelength. Dynamic mechanical analysis (DMA) was carried out on a Discovery DMA Q850 apparatus (TA Instrument) in a tension film mode. Rectangular geometry samples (ca. 0.5 mm (T) × 3 mm (W) × 20 mm (L) and a gauge length of 8 mm) were measured from –80 ℃ to 180 ℃ at a heating rate of 3 ℃ min^{-1}, a strain of 0.1% and frequency of 1 Hz. Thermogravimetric analysis (TGA) experiments were performed on a Discovery TGA550 instrument at a linear heating rate of 10 ℃ min^{-1} from 30 to 800 ℃ under N_2 atmosphere. NMR spectra were tested with a Bruker spectrometer operating at 500 MHz using DMSO-d_6 as solvent. Molecular weights were measured by gel permeation chromatography (GPC, LC-20AD XR) with DMF as eluent. The electrical properties of varying wires and sensors were tested by Keithley 2450 with a two-point measurement mode.

Electrical conductivity σ

The electrical conductivity formula is defined as:

$$R = \rho l/S \text{ and } \sigma = 1/\rho \text{, so}$$

$$\sigma = l/RS \qquad \textbf{(Equation S1)}$$

where, R is the resistance of LM/SHP wire (*e.g.*, 0.013 Ω for as-prepare wire and 0.11 Ω for healed wire), l is the length (*e.g.*, 5 cm), S is the cross-sectional area (*e.g.*, 0.38 cm^2). ρ is the electrical resistivity, σ is electrical conductivity (*e.g.*, 9.8×10^4 S m^{-1} for as-prepare wire and 1.2×10^4 S m^{-1} for healed wire),

Crosslink density (χ)

The obtained SHPs were named SHP-χ. χ is the crosslink density of SHPs, defined as:

$$\chi = \sum_{f=3}^{\infty} \frac{f}{2} C_f \qquad \textbf{(Equation S2)}$$

Where χ is the crosslink density of SHPs, f is the functionality of the reactants, and C_f is the concentration of reactant with functionality f, expressed as mole per volume of a fully cured polymer [2]. A bigger χ means a higher crosslink density.

Temperature coefficient of resistance (TCR)

The temperature coefficient of resistance (TCR) is defined as:

$$\text{TCR} = \frac{(R-R_0)/R_0}{\Delta T} \qquad \textbf{(Equation S3)}$$

where R is the resistance at varying temperatures, R_0 is the initial resistance at 27 ℃, and ΔT is the temperature change value.

Signal-to-noise ratio (SNR)

The signal-to-noise ratio (SNR) of strain sensor was calculated as [3]:

$$\mathrm{SNR} = \lg[(\frac{\Delta R}{R_0})_{\text{hand-waving}}/(\frac{\Delta R}{R_0})_{\text{tremor}}] \qquad \textbf{(Equation S4)}$$

where R_0 is the initial resistance of strain sensor, and ΔR is the resistance change value.

SUPPORTING FIGURES

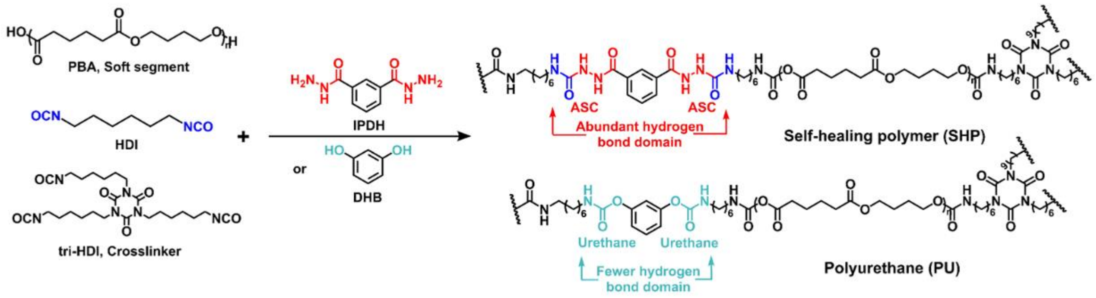

Figure S1. Synthesis routes of SHP and PU.

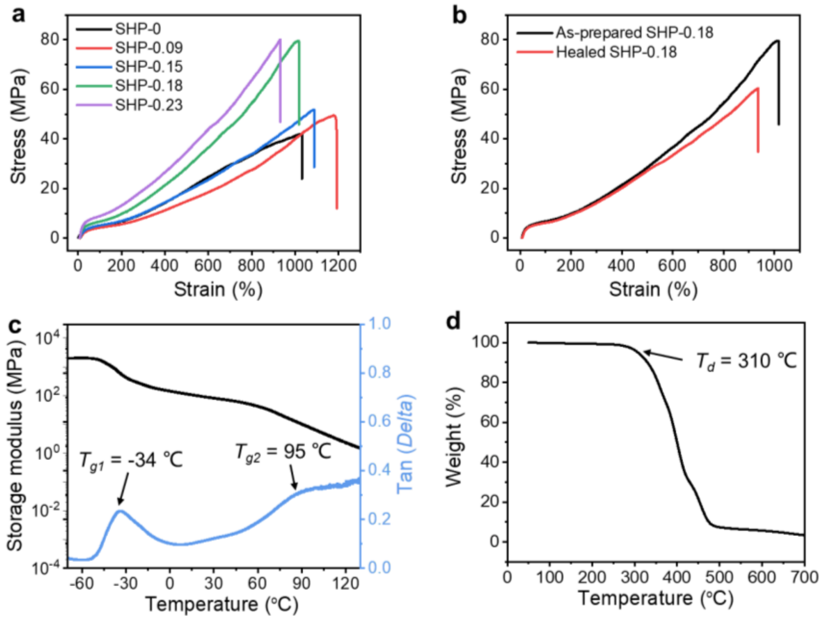

Figure S2. (a) Tensile stress-strain curves of SHP film with different crosslink densities (χ). (b) Tensile stress-strain curves of the as-prepared and healed SHP-0.18. The tensile strength of the healed SHP-0.18 was 60 MPa, and the healing efficiency was 76%. (c) Storage modulus and Tan (*Delta*) versus temperature of SHP-0.18. The glass transition temperatures (T_g) of soft and hard segments were –34 and 95 ℃, respectively. (d) TGA trace of SHP-0.18 under nitrogen atmosphere showed that a good thermal stability with 5% weight loss below 310 ℃. The thermal decomposition temperature (T_d) was 310 ℃.

The obtained SHPs were named as SHP-χ, where χ is the crosslink density of SHP (Equation S2), *i.e.*, SHP-0.18 represented a crosslink density of 0.18. Tensile stress-strain curves of SHP film material with different crosslink densities (χ) were acquired by measuring three samples in parallel to ensure the reliability of the data. The detailed data and error bars have been provided in new Table S1.

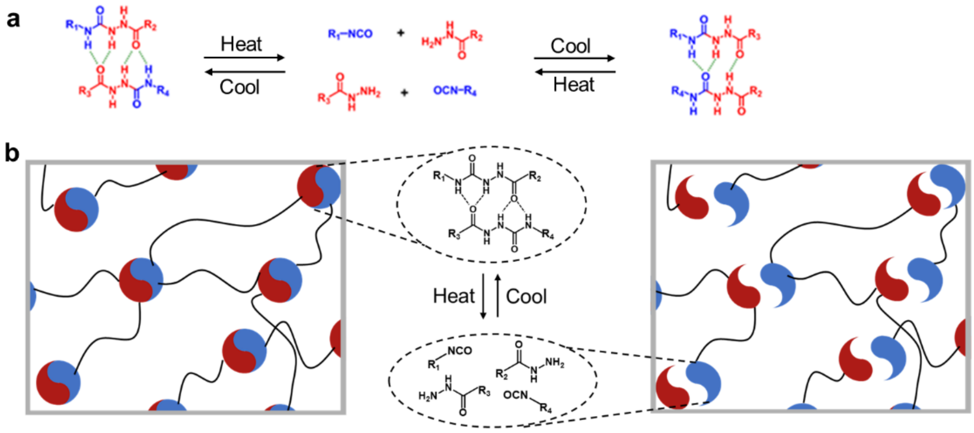

Figure S3. (a) Schematic illustration of the dynamic chemistry and hydrogen bonds of ASC moieties. (b) The dynamic dissociation/exchange reaction of ASC moieties in polymer chains.

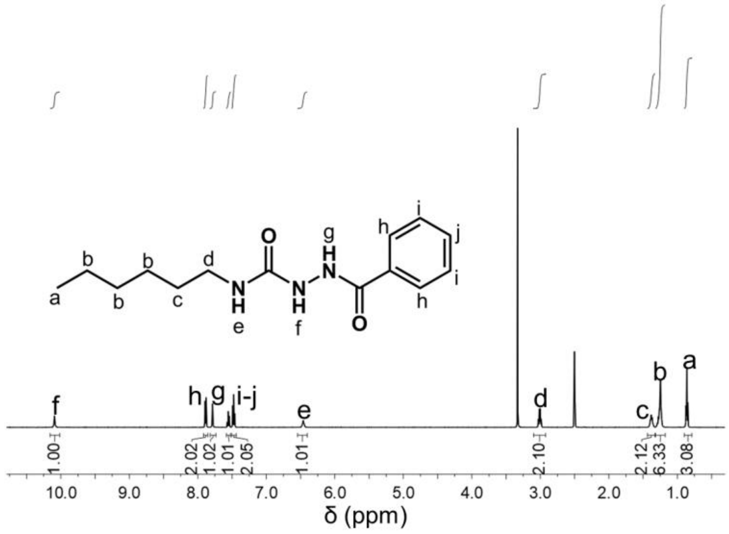

Figure S4. ^{1}H NMR spectra (500 MHz, DMSO-d_6, 298 K) of 2-benzoyl-*N*-hexylhydrazine-1-carboxamide.

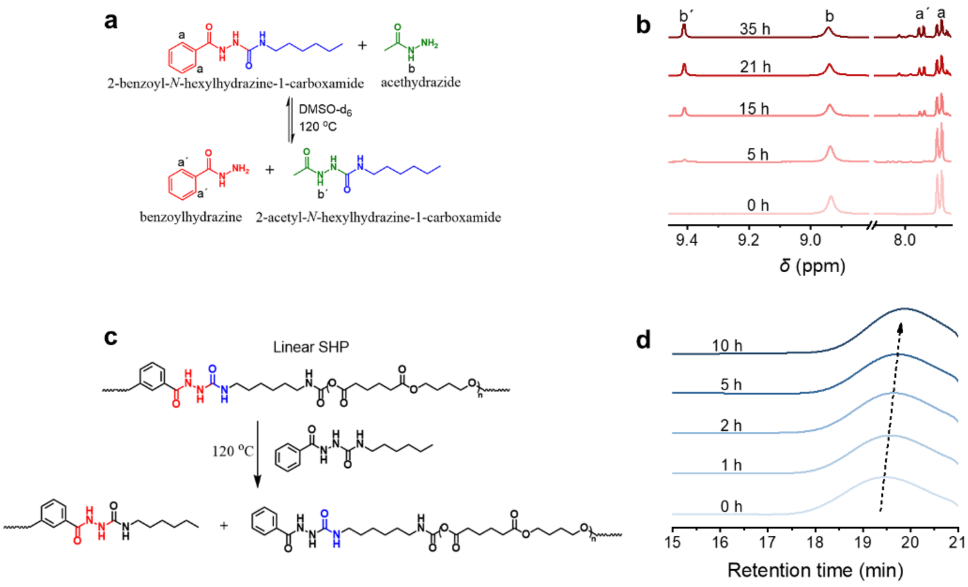

Figure S5. (a) Schematic illustration of the exchange reaction between model compound 2-benzoyl-*N*-hexylhydrazine-1-carboxamide and acethydrazide. (b) ^{1}H NMR spectra of the exchange reaction process of the model compounds, demonstrating the dynamic property of ASC moiety. (c) Schematic illustration of the exchange reaction between linear SHP and 2-acetyl-*N*-hexylhydrazine-1-carboxamide. (d) GPC spectra of the linear SHP at 120 ℃ for varying thermal treatment time of both SHP and 2-acetyl-*N*-hexylhydrazine-1-carboxamide.

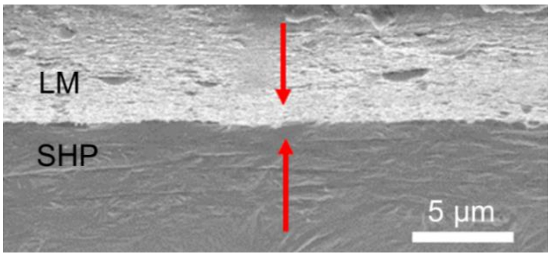

Figure S6. SEM image of the interface between SHP and LM.

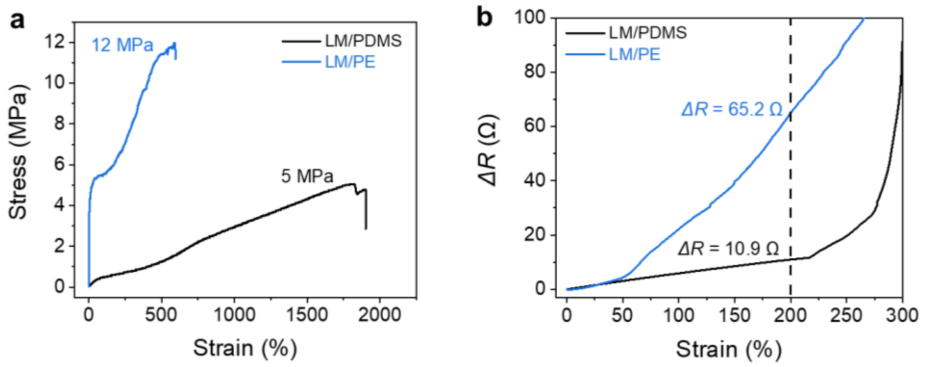

Figure S7. (a) Tensile stress-strain curves of LM/PDMS and LM/PE wires. (b) Resistance changes of LM/PDMS and LM/PE wires during stretching.

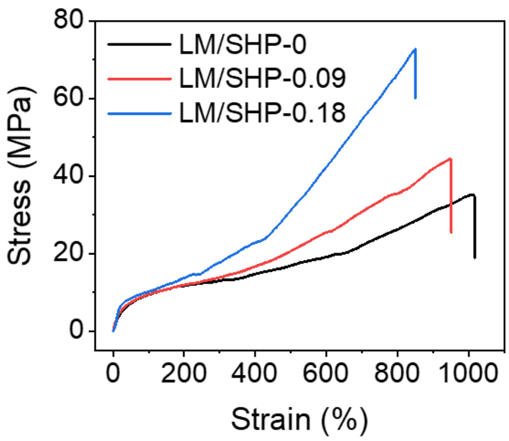

Figure S8. Tensile stress-strain curves of LM/SHP-χ wires. The tensile strength LM/SHP wires could be regulated by varying the crosslink density (χ), *e.g.*, 35 MPa of LM/SHP-0 and 73 MPa of LM/SHP-0.18, realizing a good mechanical match with common textile fibers.

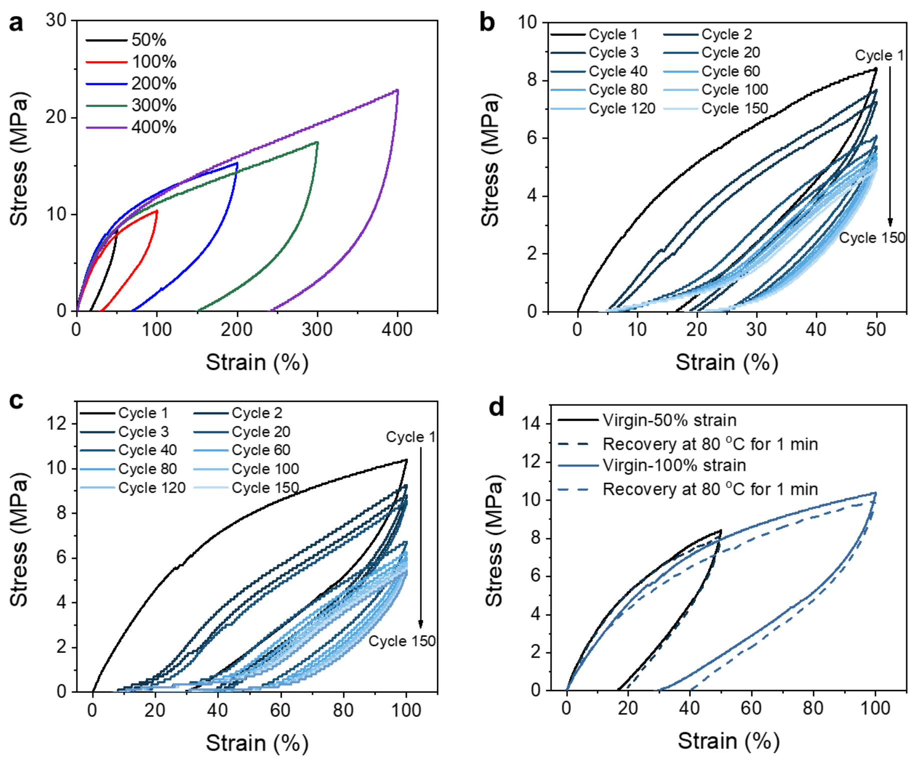

Figure S9. (a) Cyclic tensile curves of LM/SHP wires under different tensile strains (50-400%). Extended cyclic tensile experiments of LM/SHP wire at strains of (b) 50% and (c)100% for 150 cycles. (d) Elastic recovery of the LM/SHP wire after thermal treatment at 80 ℃ for 1 min.

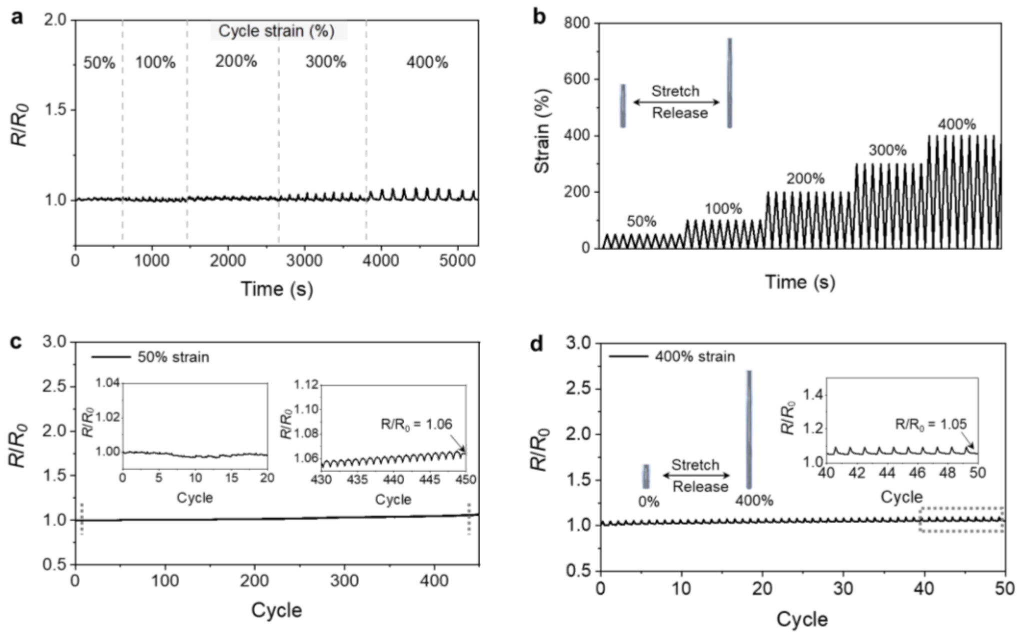

Figure S10. Variation of (a) the electrical resistances and (b) the corresponding strains of LM/SHP wires in the cyclic test. Variation of the electrical resistance in the extended cyclic test under constant strains of (c) 50% over 450 cycles and (d) 400% over 50 cycles.

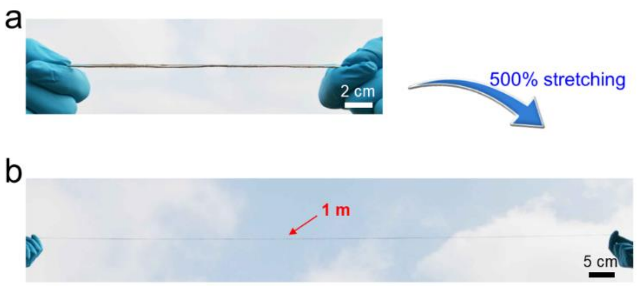

Figure S11. Photographs of (a) an as-prepared and (b) 500% stretched LM/SHP wire, respectively.

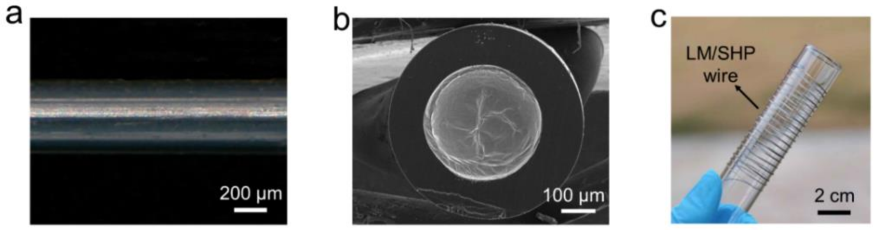

Figure S12. (a) Photograph and (b) cross-sectional SEM image of an LM/SHP wire after stretching by 500%. (c) An LM/SHP wire was wrapped on a glass tube with a radius of 15 mm.

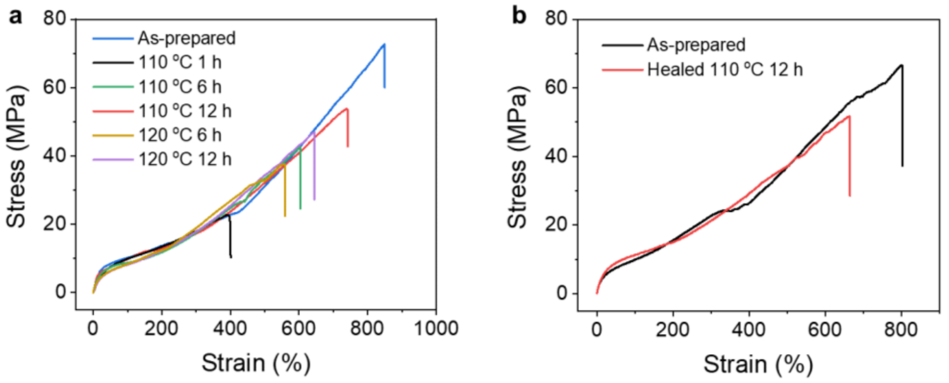

Figure S13. Stress–strain curves of the as-prepared and healed LM/SHP wires under different healing conditions with the inner diameters of (a) 0.6 and (b) 1.0 mm. The LM content could be changed by varying the inner diameters. The LM/SHP wires with an inner diameter of 1.0 mm showed a higher LM content. Photographs of LM/SHP wires with different inner diameters were illustrated in Figure S28.

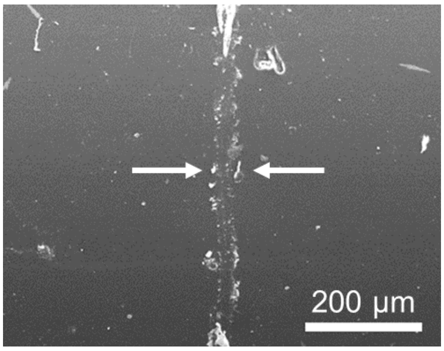

Figure S14. SEM image of the healed position of LM/SHP wire after breaking and healing.

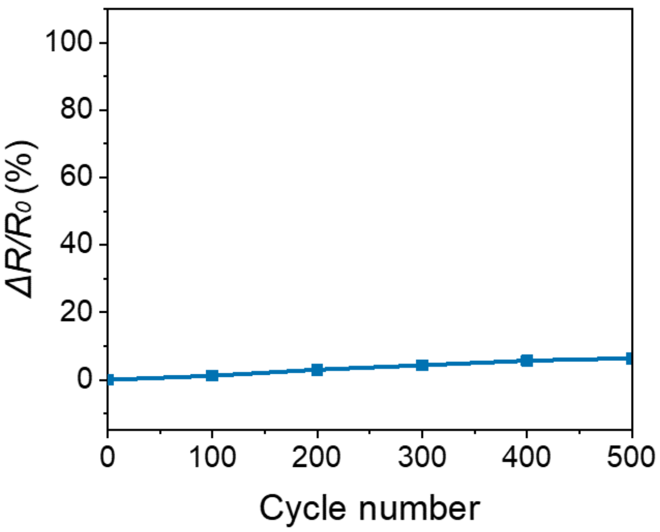

Figure S15. Electrical resistance changes of a healed LM/SHP wire during 500 stretching cycles at a 50% strain.

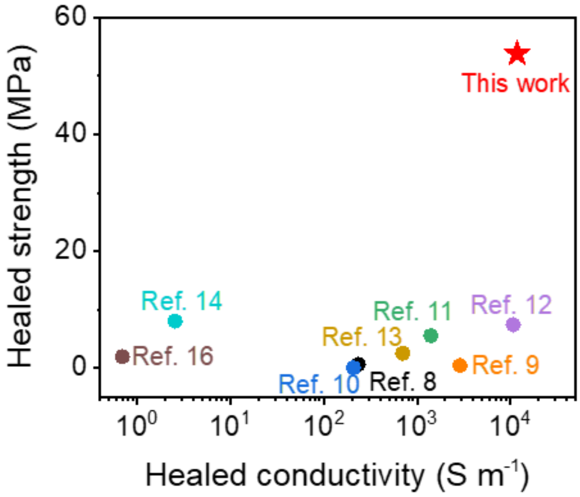

Figure S16. Comparison of the electrical conductivities and tensile strengths of the healed LM/SHP wires with previously reported self-healable wires [8-14, 16].

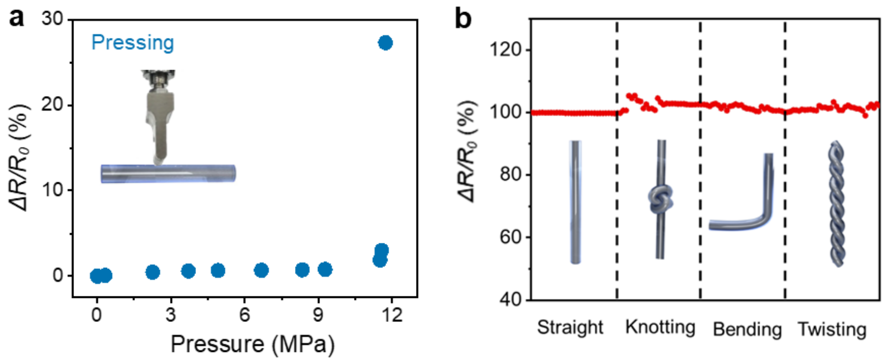

Figure S17. The electrical resistance changes of an LM/SHP wire under (a) pressing and (b) a series of other deformations.

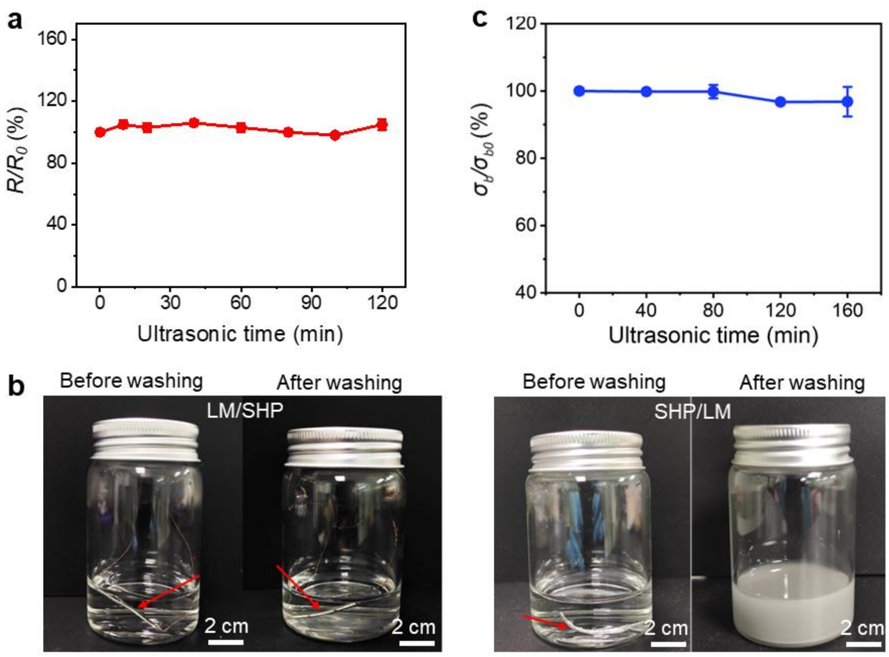

Figure S18. (a) The electrical resistance changes of LM/SHP wires under different ultrasonic washing times. (b) Photographs of the LM/SHP and SHP/LM wires before and after washing. (c) The tensile strength changes of LM/SHP wires under different ultrasonic washing times.

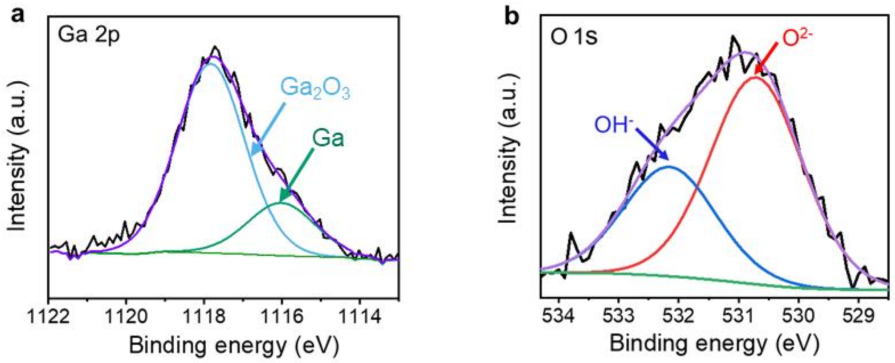

Figure S19. (a) High-resolution XPS spectra for Ga 2p of an LM droplet. The strong Ga_2O_3 signal on the surface of metallic Ga was clearly probed. (b) High-resolution XPS spectra for O 1s of an LM droplet, indicating the presence of hydroxyl groups on the surface of LM droplets.

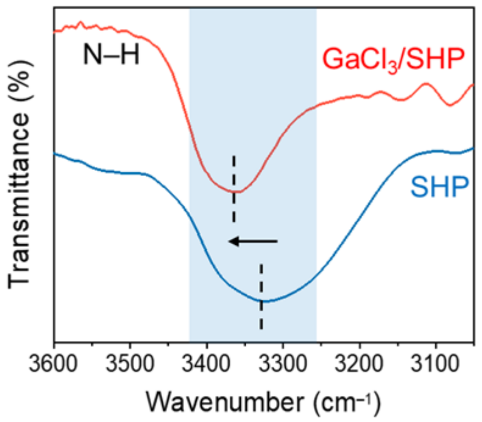

Figure S20. FTIR spectra of bare SHP and the $GaCl_3$/SHP composite in the N–H stretching region. $GaCl_3$ was added to SHP for eliminating the interference of Ga_2O_3 layer in LM. The N-H stretching vibration peak at 3,323 cm^{-1} shifted toward higher wavenumber of 3,367 cm^{-1}, implying that some N–H groups previously bound with C=O by hydrogen bond have been liberated owing to the formation of coordination bond between N-H and Ga^{3+}. Therefore, the strong hydrogen bond between N-H and the Ga_2O_3 layer might cover up the coordination interaction between N-H and Ga^{3+} (Figure 3j).

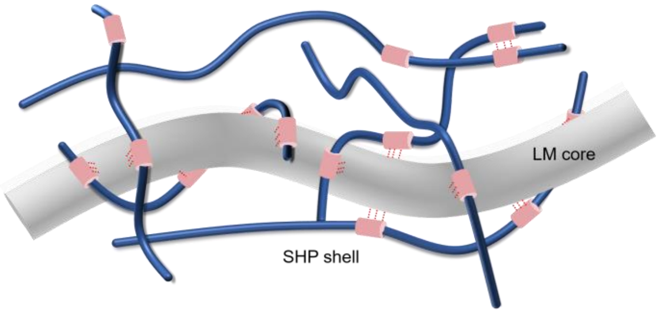

Figure S21. Schematic illustration of the interface interactions (including hydrogen and coordination bonds) between the LM core and SHP shell.

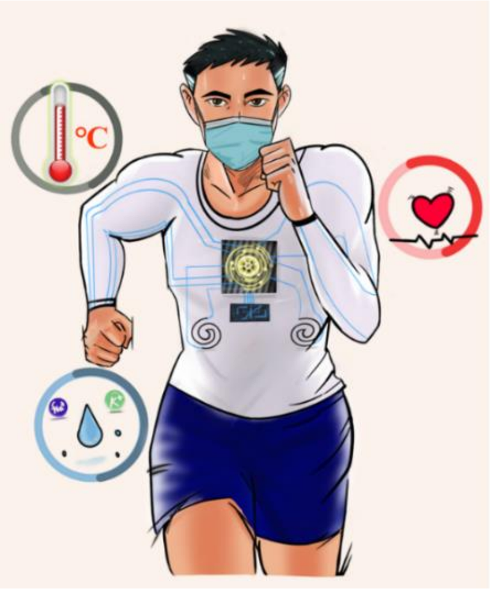

Figure S22. Schematic illustration of an integrated healthcare platform for real-time monitoring of multiple physiological signals (including temperature, pulse, and K^+ concentration).

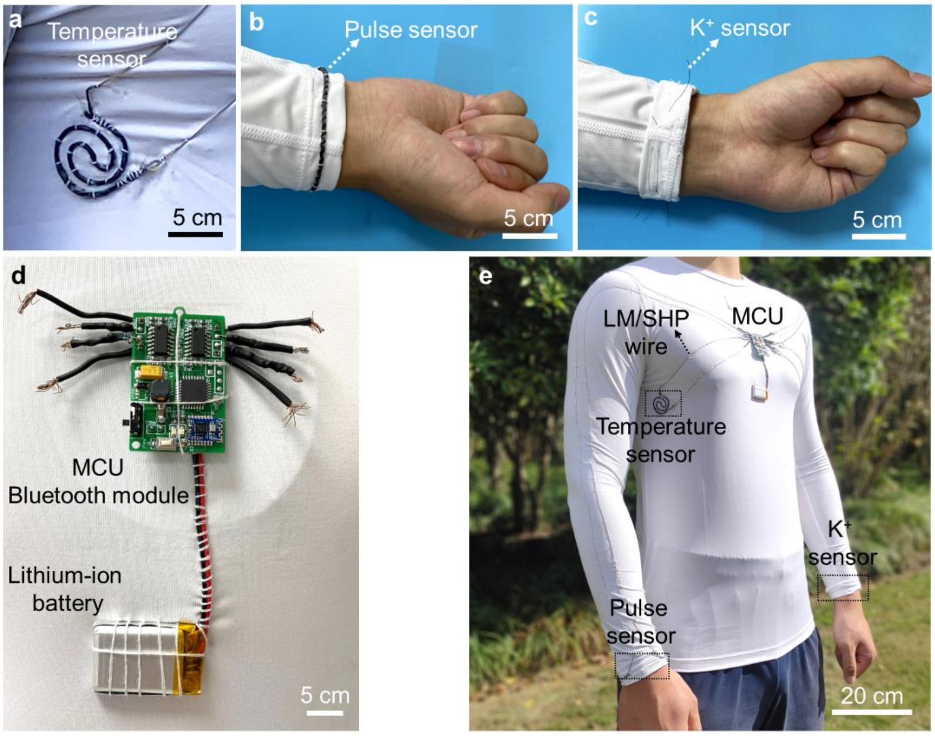

Figure S23. Photographs of the (a) temperature, (b) pulse, and (c) K^+ sensors. The temperature and pulse sensors were sewn on the outer surface of tight clothing, and the K^+ sensor was woven on the inner surface of the tight clothing. (d) Photograph of the integrated MCU, Bluetooth module, and lithium-ion battery for application demonstrations. (e) The electronic devices in (a-d) connected by the LM/SHP wires *via* a weaving process, and worn on the human body.

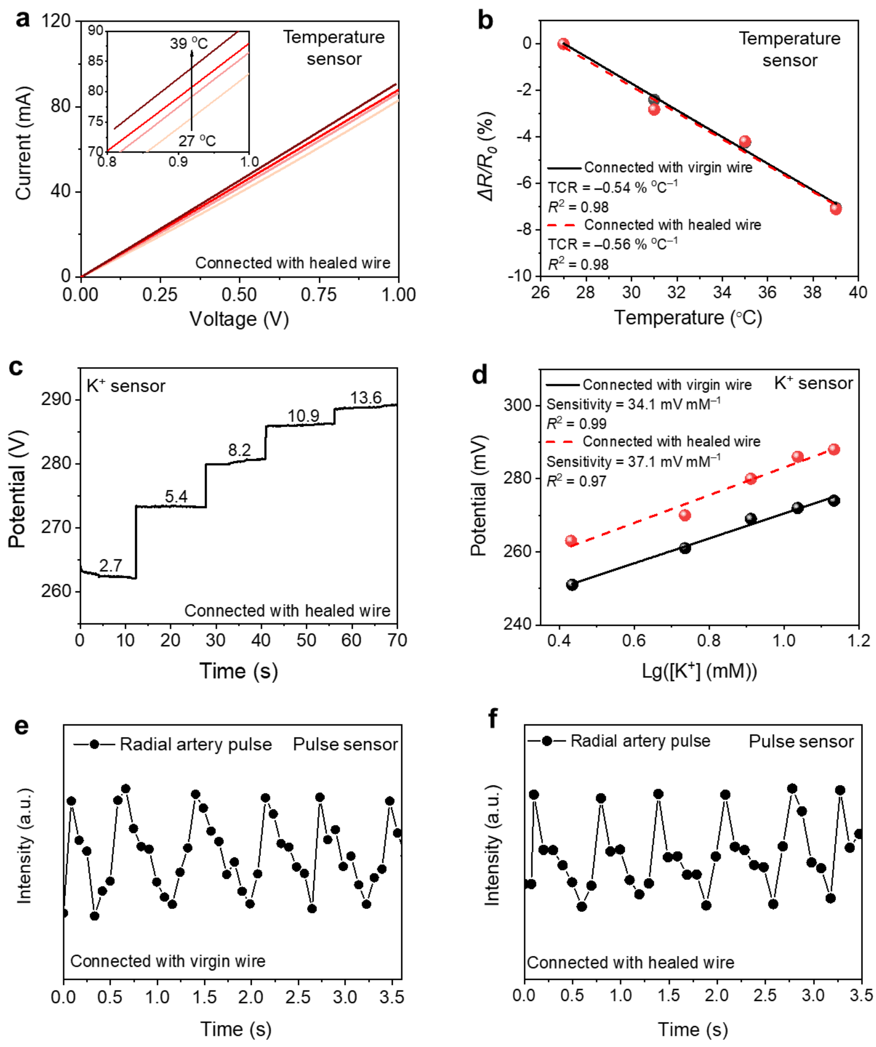

Figure S24. (a) Current-voltage curves of the wearable temperature sensor connected by a healed LM/SHP wire. (b) Temperature change values of the wearable temperature sensor interconnected with virgin and healed LM/SHP wires, which showed TCR values of –0.54 % ℃$^{-1}$ and –0.56 % ℃$^{-1}$, respectively (Equation S3). (c) Open-circuit potential response of the K^+ sensor connected by a healed LM/SHP wire in potassium chloride solutions. (d) The fitting curve of the K^+ sensor interconnected by virgin and healed LM/SHP wires, which showed high sensitivity of 34.1 mV mM^{-1} and 37.1 mV mM^{-1}, respectively. (e-f) The real-time changes of the human pulse derived from the corresponding electrical responses monitored by the pulse sensor connected by virgin and healed LM/SHP wires.

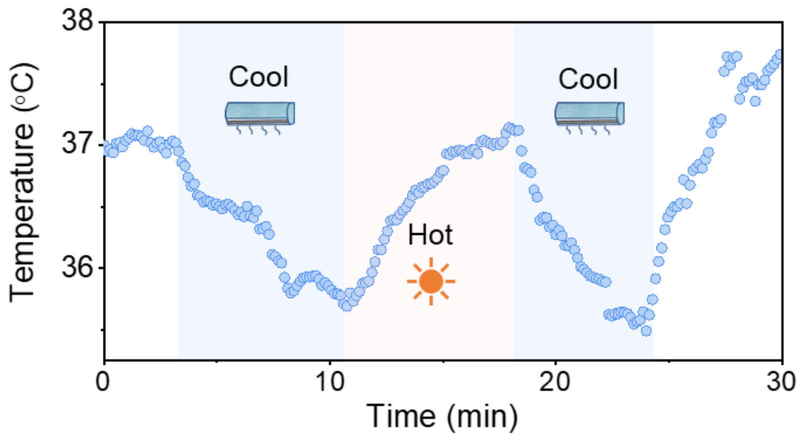

Figure S25. Real-time temperature monitoring of the human body based on the wearable healthcare platform.

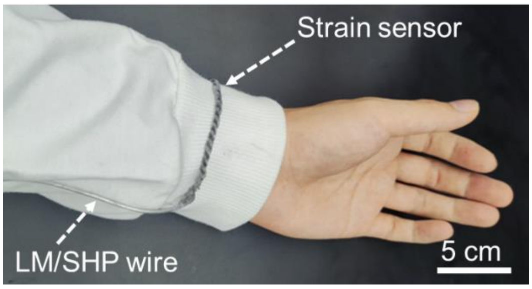

Figure S26. Photograph of the strain sensor and LM/SHP wire for precise monitoring of the hand-waving signals under a simulated case of limb tremors derived from Parkinson’s disease.

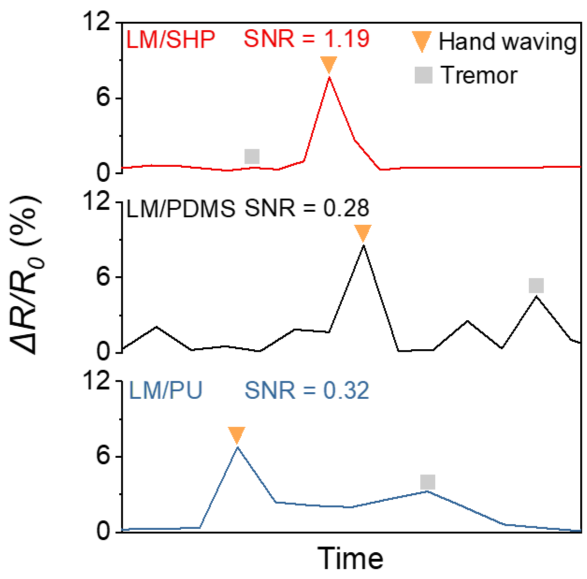

Figure S27. The SNRs of the strain sensor based on LM/SHP, LM/PDMS, and LM/PU wires under continuous limb tremor (~2.7 Hz) of Parkinson's disease. The SNRs based on LM/SHP, LM/PDMS, and LM/PU wires were calculated to be 1.19, 0.28 and 0.32, respectively.

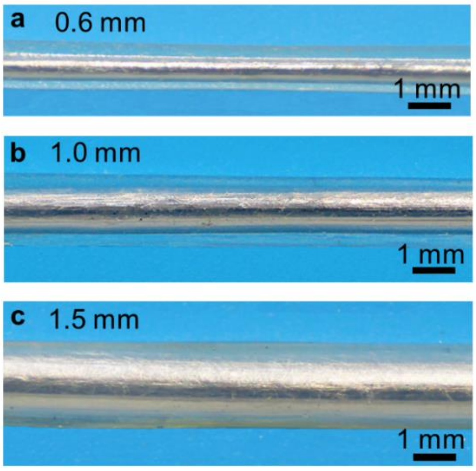

Figure S28. Photographs of LM/SHP wires with different inner diameters of (a) 0.6 mm, (b) 1.0 mm, and (c) 1.5 mm. The LM content could be changed by controlling the inner diameters.

SUPPORTING TABLES

Table S1. Mechanical properties of SHPs with different crosslink densities (χ).

Samples	χ (mmol cm^{-3})	Tensile strength (MPa)	Strain (%)	Toughness (MJ cm^{-3})
SHP- 0	0	42.0 ± 0.8	1,031.4 ± 1.5	215.3 ± 0.1
SHP-0.09	0.09	49.5 ± 1.5	1,192.4 ± 1.7	253.9 ± 1.6
SHP-0.15	0.15	51.8 ± 2.2	1,087.2 ± 0.3	248.6 ± 1.5
SHP-0.18	0.18	79.6 ± 1.1	1,017.2 ± 0.8	337.1 ± 0.1
SHP- 0.23	0.23	80.1 ± 1.3	931.1 ± 0.9	326.0 ± 1.1

Table S2. Mechanical properties of LM/SHPs wires with different crosslink densities (χ).

Samples	χ (mmol cm^{-3})	Tensile strength (MPa)	Young's modulus (MPa)	Strain (%)
LM/SHP- 0	0	35 ± 1.8	78.3 ± 0.2	1,014 ±2.6
LM/SHP-0.09	0.09	45 ± 2.0	75.1 ± 0.3	950 ± 3.2
LM/SHP-0.18	0.18	73 ± 1.6	86.2 ± 0.3	850 ± 2.0

Table S3. Mechanical properties of common textile fibers and LM/SHP wire [4, 5].

Samples	Tensile strength (MPa)	Young's modulus (MPa)	Strain (%)
Silk	33–46	587–595	15–33
Cotten	28–49	280–396	25–50
[a] PVA	58–68	278–573	15–23
[a] PA	45–73	81–302	25–58
[a] PET	60–74	609–851	18–45
LM/SHP	35–73	75–86	850–1,014

[a] PVA, PA, and PET represent polyvinyl alcohol, polyamide, and polyethylene terephthalate, respectively.

Table S4. Healing properties of LM/SHP wire with an inner diameter of 0.6 mm.

Samples		Tensile strength (MPa)	Strain (%)	Healing efficiency (%)
As-prepared	—	73 ± 1.6	850 ± 2.0	—
Healing	110 ℃, 1 h	23 ± 1.3	396 ± 1.6	32
	110 ℃, 6 h	42 ± 1.4	604 ± 1.3	58
	110 ℃, 12 h	54 ± 1.5	742 ± 1.0	74
	120 ℃, 6 h	38 ± 1.1	556 ± 0.7	52
	110 ℃, 12 h	46 ± 1.8	641 ± 1.6	64

Table S5. Healing properties of LM/SHP wires with an inner diameter of 1.0 mm.

Samples		Tensile strength (MPa)	Strain (%)	Healing efficiency (%)
As-prepared	—	67 ± 1.1	800 ± 0.9	—
Healing	110 ℃, 12 h	52 ± 1.8	664 ± 1.0	77

Table S6. Comparison of the mechanical and electrical properties of LM/SHP wire with other reported self-healable wires in previous literatures [6-16, 22].

Samples	Tensile strength (MPa)	Strain (%)	Initial conductivity (S m^{-1})	Healed conductivity (S m^{-1})	Healed Strength (MPa)	Ref.
[a] EGaIn/Reverlink	0.2	150	N/A	N/A	0.1	[6]
[a] Ga/SR	6.9	400	N/A	N/A	6.6	[7]
CNT/SHP	0.8	125	253	233	0.6	[8]
CNTs/PU/Fe_3O_4	0.7	700	2,900	2,870	0.4	[9]
P(β-CD-co-HPA)	0.1	1,000	239	207	0.04	[10]
Graphene/PU	6.0	1,000	1,700	1,400	5.5	[11]
rGO/MP	9.3	300	1.2×10^4	1.1×10^4	7.4	[12]
CNT/PAA-PEO	10.6	1,055	700	700	2.5	[13]
AgNW/PAA	7.9	1,190	2.5	2.5	8.0	[14]
[a] LM/PDMS	0.2	214	N/A	N/A	N/A	[15]
PNA/PMA	2.3	900	0.7	0.7	1.9	[16]
[a] LM/SIS-PBD	0.8	1,400	1.5×10^4	N/A	N/A	[22]
LM/SHP	72.7	850	9.8×10^4	1.2×10^4	53.8	This work

[a] The specific electrical conductivities or healed strengths were not available in these works due to the lack of electrical resistance or tensile strength data. Therefore, they were not presented in Figure S16.

Table S7. Comparison of the mechanical and electrical properties of LM/SHP wire with other reported core-shell structured LM wires in previous literatures [6, 7, 15, 17-26].

Samples		Tensile strength (MPa)	Strain (%)	Young's modulus (MPa)	Initial conductivity (S m^{-1})	Healed conductivity (S m^{-1})	Healed Strength (MPa)	Ref.
Type of core	Type of shell							
[a] EGaIn	Reverlink	0.2	150	0.2	N/A	N/A	0.1	[6]
[a] Solid Ga	SR	6.9	400	1.2–1.5	N/A	N/A	6.6	[7]
[a] GaInSn	PDMS	0.2	214	N/A	N/A	N/A	N/A	[15]
EGaIn	SEBS	14	800	N/A	3.3×10^6	N/A	N/A	[17]
[a] Solid Ga	SEBS	38	125	4–1253	N/A	N/A	N/A	[18]
GaInSn	SEBS	4.4	550	3	3.5×10^6	N/A	N/A	[19]
GaInSn	PU	1.8	480	N/A	3.5×10^6	N/A	N/A	[20]
EGaIn	PDMS	0.9	94	0.8	1.4×10^5	N/A	N/A	[21]
EGaIn	SIS-PBD	0.8	1,400	0.5–1.7	1.5×10^4	N/A	N/A	[22]
EGaIn	PVDF-HFP-TFE	7.5	1,170	3.2	4.3×10^4	N/A	N/A	[23]
GaInSn	TPU	0.1	100	N/A	5.1×10^5	N/A	N/A	[24]
EGaIn	SBS	6	2,300	N/A	6.5×10^5	N/A	N/A	[25]
[a] EGaIn	PDMS	N/A	100	N/A	N/A	N/A	N/A	[26]
GaInSn	SHP	72.7	850	86.2	9.8×10^4	1.2×10^4	53.8	This work

[a] The specific electrical conductivities or tensile strengths were not available in these works due to the lack of electrical resistance or tensile strength data. Therefore, they were not presented in Figure 2c.

Table S8. Monomer ratios of SHPs.

Samples	[a] Molar ratio	χ (mmol cm^{-3})	HDI (mmol)	PBA (mmol)	IPDH (mmol)	tri–HDI (mmol)
SHP- 0	2 : 1 : 1: 0	0	13.3	6.7	6.7	0.00
SHP-0.09	16 : 8 : 9 : 2/3	0.09	13.3	6.7	7.5	0.6
SHP-0.15	10 : 6 : 5 : 2/3	0.15	13.3	6.7	8.0	0.9
SHP-0.18	8 : 5 : 4 : 2/3	0.18	13.3	6.7	8.3	1.1
SHP- 0.23	6 : 4 : 3 : 2/3	0.23	13.3	6.7	8.9	1.5

[a] The molar ratios refer to HDI : PBA-1000 : IPDH : tri-HDI.

REFERENCES

1. Liu P, Li Y, Xu Y *et al.* Stretchable and energy-efficient heating carbon nanotube fiber by designing a hierarchically helical structure. *Small* 2018; **14**: 1702926.
2. Hill LW. Calculation of crosslink density in short chain networks. *Prog Org Coat* 1997; **31**: 235–43.
3. Chen S, Wu N, Lin S *et al.* Hierarchical elastomer tuned self-powered pressure sensor for wearable multifunctional cardiovascular electronics. *Nano Energy* 2020; **70**: 104460.
4. Grishanov S. Structure and properties of textile materials. In: Clark M. (ed.). *Handbook of textile and industrial dyeing*. Amsterdam: Elsevier, 2011; 28–63.
5. Sabir T. Fibers used for high-performance apparel. In: McLoughlin John (ed.). *High-performance apparel*. Amsterdam: Elsevier 2018; 7–32.
6. Palleau E, Reece S, Desai SC *et al.* Self-healing stretchable wires for reconfigurable circuit wiring and 3D microfluidics. *Adv Mater* 2013; **25**: 1589–92.
7. Tonazzini A, Mintchev S, Schubert B *et al.* Variable stiffness fiber with self-healing capability. *Adv Mater* 2016; **28**: 10142–8.
8. Sun H, You X, Jiang Y *et al.* Self-healable electrically conducting wires for wearable microelectronics. *Angew Chem Int Ed* 2014; **53**: 9526–31.
9. Huang Y, Huang Y, Zhu M *et al.* Magnetic-assisted, self-healable, yarn-based supercapacitor. *ACS Nano* 2015; **9**: 6242–51.
10. Li Q, Xu Z, Du X *et al.* Microfluidic-directed hydrogel fabrics based on interfibrillar self-healing effects. *Chem Mater* 2018; **30**: 8822–8.
11. Gao H, Xu J, Liu S *et al.* Stretchable, self-healable integrated conductor based on mechanical reinforced graphene/polyurethane composites. *J Colloid Interface Sci* 2021; **597**: 393–400.
12. Niu P, Bao N, Zhao H *et al.* Room-temperature self-healing elastomer-graphene composite conducting wires with superior strength for stretchable electronics. *Compos Sci Technol* 2022; **219**: 109261.
13. Li J, Sun J, Wu D *et al.* Functionalization-directed stabilization of hydrogen-bonded polymer complex fibers: elasticity and conductivity. *Adv Fiber Mater* 2019; **1**: 71–81.
14. Wu Y-Y, Chen H, Zhang F *et al.* Stretchable and self-healing conductive fibers from hierarchical silver nanowires-assembled network. *Nano Res* 2023; doi: 10.1007/s12274-023-5797-5.
15. Zhou Y, Zhu Y, Hu ZA *et al.* Liquid metal‐based self‐healable and elastic conductive fiber in complex operating conditions. *Energy Environ Mater* 2022; **6:** e12448.
16. Shuai L, Guo ZH, Zhang P *et al.* Stretchable, self-healing, conductive hydrogel fibers for strain sensing and triboelectric energy-harvesting smart textiles. *Nano*

Energy 2020; **78**: 105389.
17. Zhu S, So JH, Mays R *et al.* Ultrastretchable fibers with metallic conductivity using a liquid metal alloy core. *Adv Funct Mater* 2013; **23**: 2308–14.
18. Park S, Baugh N, Shah HK *et al.* Ultrastretchable elastic shape memory fibers with electrical conductivity. *Adv Sci* 2019; **6**: 1901579.
19. Qu Y, Nguyen-Dang T, Page AG *et al.* Superelastic multimaterial electronic and photonic fibers and devices via thermal drawing. *Adv Mater* 2018; **30**: 1707251.
20. Yu Y, Guo J, Ma B *et al.* Liquid metal-integrated ultra-elastic conductive microfibers from microfluidics for wearable electronics. *Science Bulletin* 2020; **65**: 1752–9.
21. Markvicka EJ, Bartlett MD, Huang X *et al.* An autonomously electrically self-healing liquid metal-elastomer composite for robust soft-matter robotics and electronics. *Nat Mater* 2018; **17**: 618–24.
22. Tutika R, Haque AT, Bartlett MD. Self-healing liquid metal composite for reconfigurable and recyclable soft electronics. *Commun Mater* 2021; **2**: 64.
23. Zheng L, Zhu M, Wu B *et al.* Conductance-stable liquid metal sheath-core microfibers for stretchy smart fabrics and self-powered sensing. *Sci Adv* 2021; **7**: eabg4041.
24. Xiong Y, Xiao J, Chen J *et al.* A multifunctional hollow TPU fiber filled with liquid metal exhibiting fast electrothermal deformation and recovery. *Soft Matter* 2021; **17**: 10016–24.
25. Zhou N, Jiang B, He X *et al.* A superstretchable and ultrastable liquid metal-elastomer wire for soft electronic devices. *ACS Appl Mater Interfaces* 2021; **13**: 19254–62.
26. Krisnadi F, Nguyen LL, Ankit *et al.* Directed assembly of liquid metal–elastomer conductors for stretchable and self-healing electronics. *Adv Mater* 2020; **32**: 2001642.
